# The Obesity Paradox Reconsidered: Evidence from a Multicenter Romanian Hemodialysis Cohort

**DOI:** 10.3390/jcm15010357

**Published:** 2026-01-03

**Authors:** Alexandru Catalin Motofelea, Radu Pecingina, Nicu Olariu, Luciana Marc, Lazar Chisavu, Flaviu Bob, Adelina Mihaescu, Adrian Apostol, Oana Schiller, Nadica Motofelea, Adalbert Schiller

**Affiliations:** 1 Department of Doctoral Studies, “Victor Babes” University of Medicine and Pharmacy Timisoara, Eftimie Murgu Square No. 2, 300041 Timisoara, Romania; alexandru.motofelea@umft.ro (A.C.M.); nadica.motofelea@umft.ro (N.M.); 2 Centre for Molecular Research in Nephrology and Vascular Disease/MOL-NEPHRO-VASC, “Victor Babes” University of Medicine and Pharmacy Timisoara, 300041 Timisoara, Romania; marc.luciana@umft.ro (L.M.); chisavu.lazar@umft.ro (L.C.); bob.flaviu@umft.ro (F.B.); mihaescu.adelina@umft.ro (A.M.); schiller.adalbert@umft.ro (A.S.); 3 Department VII, Internal Medicine II, Division of Nephrology, “Victor Babes” University of Medicine and Pharmacy Timisoara, 300041 Timisoara, Romania; 4 County Emergency Hospital, L. Rebreanu Street, Nr. 156, 300723 Timisoara, Romania; 5 Department VII, Internal Medicine II, Discipline of Cardiology, “Victor Babes” University of Medicine and Pharmacy Timisoara, Eftimie Murgu Square, No. 2, 300041 Timisoara, Romania; apostol.adrian@umft.ro; 6 Department of Cardiology, Pius Brinzeu Clinical Emergency County Hospital Timisoara, 300736 Timisoara, Romania; 7 B Braun Avitum Dialysis Centre, 300417 Timisoara, Romania; oana.leo67@gmail.com; 8 Department of Obstetrics and Gynecology, “Victor Babes” University of Medicine and Pharmacy Timisoara, Eftimie Murgu Square No. 2, 300041 Timisoara, Romania

**Keywords:** hemodialysis, obesity paradox, body mass index, chronic kidney disease, end-stage kidney disease, all-cause mortality, cardiometabolic risk

## Abstract

**Background and Objectives**: The obesity paradox in maintenance hemodialysis (MHD) patients (better survival of obese as compared to non-obese patients in MHD) remains controversial, with many published papers supporting the idea that higher BMI is protective. Data from Eastern Europe, in particular from the elderly population on hemodialysis, are limited. The aim of this study was to describe the distribution of body weight status and cardiometabolic comorbidities and to evaluate the association of BMI categories with all-cause mortality in a multi-center Romanian hemodialysis cohort. **Materials and Methods**: We conducted a retrospective cohort study of 679 patients with end-stage kidney disease (ESKD) undergoing maintenance haemodialysis in eight Romanian centers. All patients received thrice-weekly treatments (≥4 h/session) using high-flux dialysers. Baseline demographic, clinical, laboratory, and echocardiographic data were extracted from dialysis records. Survival across BMI groups was assessed using Kaplan–Meier curves and the log-rank test. Cox proportional hazards models were used to estimate hazard ratios (HRs) for all-cause mortality, with normal weight as the reference category. Multivariable models incorporated progressive adjustment for age, sex, dialysis vintage, diabetes, major cardiovascular comorbidities, and ESKD-related factors, including anemia parameters and CKD–mineral and bone disorder (CKD-MBD) markers. **Results**: A total of 679 haemodialysis patients were included (mean age 57.2 ± 12.9 years; 59.1% male); 52.7% were normal weight, 28.9% overweight, and 18.4% obese. During follow-up, 360 patients (53.0%) died, with similar crude mortality across BMI groups (normal weight 51.7%, overweight 55.1%, obese 53.6%; *p* > 0.05). In univariate Cox analyses, older age, obesity, hypoalbuminaemia, elevated CRP, hyperphosphataemia, peripheral and cerebrovascular disease, diabetes, low dialysis adequacy (eKt/V < 1.2), and lower ultrafiltration were associated with higher mortality, whereas preserved LVEF (≥50%) was protective. In multivariable analyses, independent predictors of mortality included older age (HR 1.042 per year, *p* < 0.001), obesity (HR 1.411, *p* = 0.045), elevated CRP (HR 1.781, *p* < 0.001), diabetes (HR 1.775, *p* < 0.001), inadequate dialysis dose (eKt/V < 1.2; HR 1.343, *p* = 0.029), and preserved LVEF remained protective (HR 0.665, *p* = 0.013). The Kaplan–Meier analysis showed significantly lower survival with increasing BMI: median survival was 7.56 years in normal-weight patients, 4.56 years in overweight patients, and 3.92 years in obese individuals (log-rank *p* < 0.05). **Conclusions**: In this Romanian cohort of multicenter hemodialysis patients, obesity as measured by BMI was associated with an increased incidence of all-cause mortality, while overweight did not confer a clear survival advantage over normal weight. These findings call into question the classic hemodialysis obesity paradox and support a more cautious interpretation of the increased BMI.

## 1. Introduction

Chronic kidney disease (CKD) and obesity are becoming major health issues all over the world, having a positive epidemiology and generating high cardiovascular and mortality risk. Obesity increases the risk of coronary heart disease by 2–3-fold, of heart failure by 4-fold, and the risk of stroke by 1.5–2-fold [1]. Obesity also predicts the onset of CKD in the general population [2] and increases the rate of decline of kidney function and mortality in CKD [3]. The prevalence of obesity is higher in CKD as compared to the general population (at least in the USA), and it presents an increasing trend all over the CKD spectrum (including predialysis, hemodialysis, and kidney transplantation) [4]. The body mass index (BMI), in clinical settings, is the most frequently used tool to assess overweight and obesity in the general population as well as in CKD and ESKD. Though having well-known limitations (influenced by extracellular volume expansion), BMI offers a fairly good reflection of total body fat in patients with CKD and ESKD [4,5].

Since 1999, the obesity paradox in HD patients has been described (i.e., lower mortality rate in obese HD patients as compared to normal body weight ones, the reverse of what was registered in the general population) [6,7]. The obesity paradox has been attributed to malnutrition–inflammation syndrome (the paradox seems to function only in HD patients with normal or increased muscle mass [8], hemodynamic stability status, changed cytokine synthesis and circulation, and so on [9]). Some confirmatory studies have also been published [10,11,12]. In recent years the obesity paradox in HD patients has been more and more frequently challenged. A meta-analysis showing substantial heterogeneity has also been published [13,14,15].

During these 20 years since obesity paradox in HD patients was identified, many changes occurred in the management of CKD patients: the average age of patients admitted to HD therapy has increased, more DM patients are included in HD treatment, and prophylactic approaches in predialysis CKD have been implemented (iSGLT2, GLP1 RA, and MRA), so the patients admitted to HD therapy present a modified risk profile and these data are more evident in emerging countries. Guidelines implementations in clinical practice are dependent on economic development, political, and cultural influences. Therefore, the aim of this paper is to assess, by a multicenter retrospective cohort study (representative for the HD-treated population in Romania), the effects of overweight and obesity on survival of HD patients.

## 2. Materials and Methods

A total of 679 adult patients undergoing maintenance hemodialysis were included in this study from eight dialysis centers across Romania, covering all historical regions of the country. Inclusion criteria were age ≥ 18 years, treatment with thrice-weekly hemodialysis (≥12 h per week) using high-flux, high-surface-area dialyzers, and clinical stability at the time of enrollment. The KDIGO guidelines were followed when evaluating treatment and monitoring anemia and chronic kidney disease—mineral and bone disorder (CKD-MBD) [16,17]. Patients were excluded if they had a documented diagnosis of active or previous malignancy; were younger than 18 years; or lacked essential clinical, laboratory, or follow-up data required for survival analyses.

**Figure 1:**
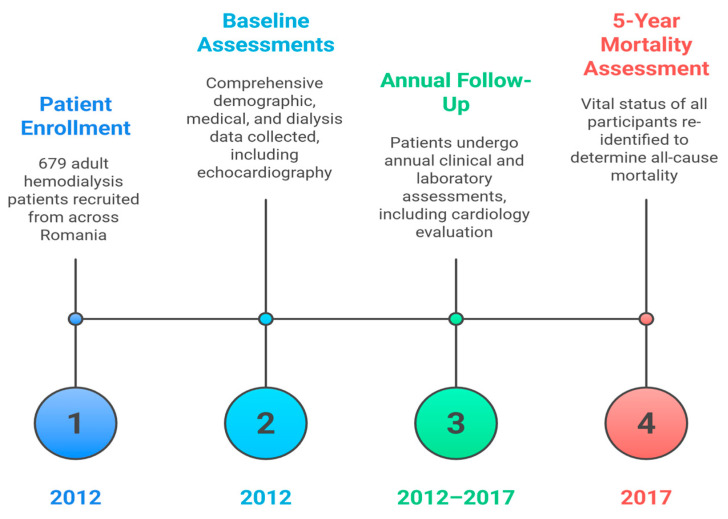
Study Timeline and Follow-Up Scheme.

Patients’ dialysis files were used to obtain personal information, medical history, and HD treatment parameters. The patients underwent yearly cardiology assessment in addition to the clinical evaluation. In compliance with the guidelines of the European Association of Cardiovascular Imaging (EACI), a two-dimensional, M-mode continuous, and pulse Doppler echocardiography was carried out between the second and third hours of the dialysis session by the same operator for each cohort using comparable equipment (to avoid inter-observer differences). The operator recorded the presence and quantity of heart valve calcifications, measured left ventricular hypertrophy, and evaluated the left ventricular ejection fraction (LVEF) using the Simpson method. Body mass index (BMI) categories were defined according to the World Health Organization classification: normal weight (18.5–24.9 kg/m^2^), overweight (25–29.9 kg/m^2^), and obesity (≥30 kg/m^2^). In line with these thresholds, our cohort was stratified into normal weight (<25 kg/m^2^), overweight (25–29.9 kg/m^2^), and obese (≥30 kg/m^2^) groups. Mortality was followed up for 5 years. The 5-year survivors from the 2012 cohort were identified in 2017, and their data changes were assessed. The study timeline and follow-up scheme are summarized in Figure 1.

The Ethics Committee of each dialysis center approved the study, and all patients provided written informed consent permitting the investigators to use their personal data for scientific purposes. The study was conducted in accordance with the Ethics Code of the World Medical Association, approval no. 80/10.11.2021, revised in 2024.

### Statistical Analysis

For numerical variables with a Gaussian distribution, the data are displayed as mean ± standard deviation; for numerical variables with a non-Gaussian distribution, they are displayed as median and interquartile range, respectively, as a percentage of the sub-group total and the total number of individuals. The Shapiro–Wilk test and Levene’s test were used to check the distribution of continuous variables for normality and equality of variances. Cox regression, both univariable and multivariable, was used to calculate adjusted risk estimates for all-cause death. A *p*-value of 0.05 was used in this investigation as the cutoff point for statistical significance. R studio version 4.4.3 was utilized for data analysis.

## 3. Results

After exclusions of patients with known cancer diagnosis, 679 patients undergoing maintenance hemodialysis were included in the analyses. Table 1 summarizes the baseline demographic, clinical, and biochemical characteristics across BMI categories. Patients classified according to BMI were of normal weight in 52.7% of cases (mean BMI 21.5 ± 2.3 kg/m^2^), overweight in 28.9% (mean BMI 27.6 ± 1.4 kg/m^2^), and obese in 18.4% (mean BMI 34.7 ± 4.1 kg/m^2^). Mean age of the cohort was 57.2 years, and 59.1% were male. No meaningful differences were observed for mortality across BMI categories.

Age differed significantly across BMI groups (*p* = 0.002), with the normal-weight patients being younger than both overweight and obese individuals. Sex distribution did not differ significantly.

Several dialysis-related characteristics varied by BMI groups. Dialysis vintage decreased progressively with increasing BMI (*p* < 0.001), with the longest duration in the normal-weight patients and the shortest in obese individuals. Ultrafiltration volume per session was significantly higher in both the overweight and obese patients compared with the normal-weight group (*p* = 0.032 and *p* = 0.018). Dialysis adequacy (eKt/V) was significantly lower in the obese patients compared with the other two groups (*p* = 0.040 and *p* < 0.001).

Biochemical markers also demonstrated variations with BMI groups. Ferritin and transferrin saturation were significantly higher in the normal-weight patients compared with overweight and obese individuals (all *p* ≤ 0.009). CRP was significantly higher in overweight compared with the normal-weight patients (*p* = 0.034), whereas no meaningful differences were observed for albumin, hemoglobin, bicarbonate, calcium, or iPTH.

Mineral metabolism parameters varied across BMI strata. The obese patients exhibited higher serum phosphate and calcium–phosphate product values compared with the normal-weight patients (*p* = 0.002).

Cardiovascular comorbidities also differed by BMI groups. Coronary artery disease was significantly more prevalent among the overweight and obese patients than among the normal-weight individuals (*p* = 0.001 and *p* = 0.011). Peripheral vascular disease occurred more frequently in the obese patients (*p* = 0.045). No significant differences were observed for heart valve calcifications, left ventricular hypertrophy, or left ventricular ejection fraction.

Metabolic and infectious comorbidities varied substantially. Diabetes mellitus was more common among overweight and obese individuals compared with the normal-weight patients (*p* = 0.001 and *p* < 0.001). Conversely, hepatitis C was significantly more prevalent in the normal-weight patients than in the overweight or obese groups (*p* = 0.035 and *p* < 0.001) (Table 1).

### Survival Analysis

A Cox proportional hazards regression model was used to assess the association between clinical and dialysis-related variables and all-cause mortality in the hemodialysis cohort. In univariate analyses, older age, obesity, hypoalbuminemia, elevated CRP, phosphate above target, vascular comorbidities (peripheral and cerebrovascular disease), diabetes, low dialysis dose (eKt/V < 1.2), and lower ultrafiltration were all significantly associated with mortality, whereas patients with preserved LVEF had a better survival (Table 2).

In the multivariable model, which simultaneously adjusted for age, sex, weight category, LVEF, guideline-based laboratory variables, comorbidities, and dialysis parameters, several factors remained independently associated with outcome. Using normal weight as the reference, obesity remained significantly associated with higher mortality (HR 1.411, 95% CI 1.008–1.976, *p* = 0.045), whereas overweight was not (HR 1.022, 95% CI 0.781–1.339, *p* = 0.873). Each additional year of age was associated with an approximately 4% increase in the hazard of death (HR 1.042, 95% CI 1.031–1.052, *p* < 0.001). Regarding cardiac function, preserved LVEF (≥50%) was independently associated with better survival (HR 0.665, 95% CI 0.482–0.918, *p* = 0.013), while mildly reduced LVEF (41–49%) did not reach statistical significance after adjustment. Among biochemical markers, elevated CRP > 0.5 mg/dL was strongly associated with mortality (HR 1.781, 95% CI 1.289–2.461, *p* < 0.001), and ferritin values outside 200–500 ng/mL showed a modest inverse association with risk (HR 0.749, 95% CI 0.589–0.952, *p* = 0.018). Classical dialysis targets such as hypoalbuminemia, low hemoglobin, hyperphosphatasemia, and abnormal iPTH were significant only in univariate analyses and lost significance in the fully adjusted model.

Vascular comorbidities (coronary, peripheral, and cerebrovascular disease) were associated with worse survival in univariate models, but their effects were attenuated and no longer significant after adjustment, suggesting confounding by age and other factors. In contrast, diabetes mellitus remained a strong independent predictor of mortality (HR 1.775, 95% CI 1.338–2.355, *p* < 0.001). Finally, inadequate dialysis dose (eKt/V < 1.2) was independently associated with higher mortality (HR 1.343, 95% CI 1.031–1.749, *p* = 0.029), whereas higher ultrafiltration (>500 mL/h) showed only a non-significant trend towards lower risk (HR 0.702, 95% CI 0.481–1.026, *p* = 0.067) (Table 2).

The Kaplan–Meier survival analysis according to BMI categories over time on dialysis (dialysis vintage) revealed significantly lower survival probabilities in obese and overweight patients compared with the normal-weight individuals. The log-rank test confirmed a statistically significant difference between the three groups (*p* < 0.05).

**Figure 2:**
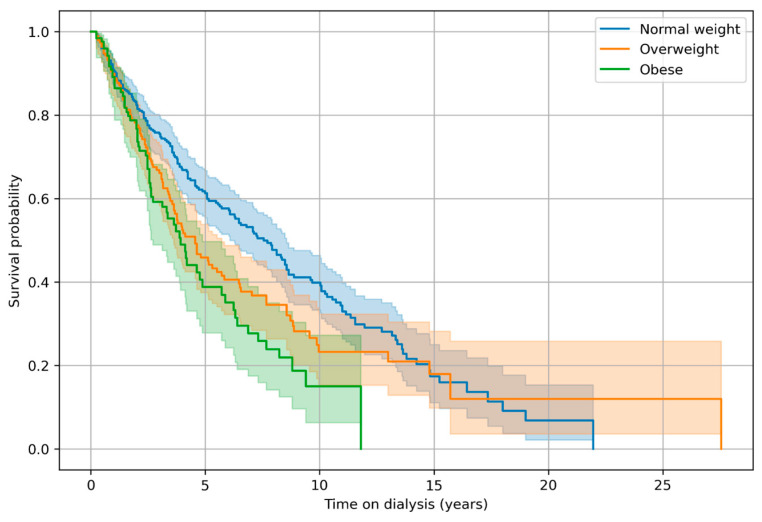
Kaplan–Meyer showing the survival of the patients with ponderal status.

The survival outcomes demonstrated marked differences across weight categories in this hemodialysis cohort of 679 patients. Among the normal-weight patients (358 individuals), 185 deaths occurred during follow-up, with a median survival time on dialysis of 7.56 years. In contrast, the overweight group of 195 patients experienced 107 deaths and a shorter median dialysis vintage until death of 4.56 years, representing a reduction of approximately three years compared with the normal-weight patients. The obese category, comprising 125 patients with 67 deaths, exhibited the poorest survival profile, with a median time on dialysis of only 3.92 years, nearly half that observed in the normal-weight group (Figure 2).

## 4. Discussion

Currently, obesity is regarded as a chronic disease, and obesity-related problems in patients with chronic kidney disease have attracted increasing attention [18,19]. In 2002, the prevalence of obesity in incident ESKD in the U.S. Renal Data System was reported to be 29% [20]. Data from several European countries indicated a lower proportion of obese patients on dialysis compared with the U.S., ranging from 10% to 12% [21]. The trends were positive both in the US and in Europe, and the average BMI at initiation in HD patients was also increasing. In our Romanian hemodialysis cohort, 28.9% of patients were overweight, and 18.4% were obese according to BMI categories, proportions broadly comparable to those reported in other high- and middle-income countries. We could not systematically assess abdominal obesity using waist circumference, which is an important limitation of our analysis. Previous papers have shown that BMI-based definitions of obesity may underestimate the burden of excess adiposity in HD patients and fail to capture central fat accumulation, which is more strongly related to cardiometabolic risk [22,23]. By analogy, it is plausible that our HD patients with “normal” or “overweight” BMI might still carry a higher burden of visceral adiposity, which we could not directly quantify.

BMI is an imperfect surrogate for adiposity in end-stage renal disease, as it fails to capture fat distribution or distinguish fat from lean mass. Accordingly, measures of abdominal obesity, such as waist circumference, have been shown to be stronger predictors of all-cause and cardiovascular mortality than BMI [24]. In a large nationally representative Korean hemodialysis cohort of 18,699 patients, higher BMI was inversely associated with mortality, whereas increased waist circumference reflecting central adiposity was independently associated with higher all-cause mortality, indicating that fat distribution rather than overall body size drives adverse outcomes [25].

Importantly, the prognostic significance of higher body size in dialysis appears to be largely driven by muscle mass. In hemodialysis, higher serum creatinine, a surrogate of muscle mass, has been consistently associated with improved survival, and similar associations have been observed in peritoneal dialysis [26]. In a cohort of 10,896 PD patients, higher baseline serum creatinine was progressively protective (HR 0.64 for ≥14.0 mg/dL), whereas an early decline in creatinine predicted increased mortality, suggesting that muscle mass–survival relationships extend beyond dialysis modality, albeit with attenuation early after treatment initiation [27].

Prospective data in peritoneal dialysis further confirm the adverse prognostic impact of sarcopenic obesity. In a cohort of 223 PD patients followed for a median of 51.6 months, patients with sarcopenic obesity (11.2%) had significantly lower survival, with a more than two-fold higher mortality risk (HR 2.19, 95% CI 1.01–4.74) compared with nonsarcopenic, non-obese patients. These findings underscore that the coexistence of muscle depletion and excess adiposity confers substantial risk, independent of dialysis modality [28].

Chronic inflammation strongly influences mortality in hemodialysis and may modify obesity–survival associations. In a multinational cohort of 5061 patients, C-reactive protein was independently associated with mortality; outperformed other inflammatory markers; and was positively associated with male sex, older age, and higher BMI, highlighting inflammatory burden as a key contributor to adverse outcomes across BMI categories [29].

Because body composition data were unavailable, we could not distinguish between robust obesity and sarcopenic obesity. However, prior studies using CT-based body composition assessment have demonstrated that sarcopenic obesity—characterized by low skeletal muscle mass and high fat mass—is strongly associated with increased mortality in hemodialysis patients, with concomitant myosteatosis further amplifying risk, suggesting that impaired muscle mass or muscle quality may partly underlie the adverse prognosis associated with higher BMI in our cohort [30].

Prospective data using repeated bioimpedance assessments show that longitudinal body composition changes are stronger predictors of mortality than baseline measures in hemodialysis patients. In a cohort of 340 patients, loss of lean tissue index (HR 3.40) and gain of fat tissue index (HR 4.06) were independently associated with all-cause mortality, while low baseline LTI lost significance after adjustment for comorbidities; patients with concomitant LTI loss and FTI gain had a more than five-fold higher mortality risk (HR 5.34). These findings support the interpretation that adverse outcomes attributed to higher BMI may reflect unfavorable muscle–fat redistribution rather than body weight itself [31].

Large registry data further support the prognostic relevance of muscle mass in hemodialysis. In an analysis of 26,625 adult patients from the Korean Society of Nephrology registry, a low lean body mass index (LBMI ≤ 15.55) was consistently associated with reduced survival across all age groups, with the strongest effect observed in older patients. Notably, in patients aged ≥50 years, low LBMI had a greater impact on mortality than diabetes, underscoring the dominant role of muscle depletion in long-term outcomes [32]. Data from elderly populations further highlight the close interplay between nutritional risk, inflammation, and adverse body composition in hemodialysis. In a case–control study of 84 elderly HD patients and 84 matched controls, HD patients had a markedly higher prevalence of nutritional risk by GNRI (32.1% vs. 6.0%), lower phase angle and serum albumin, higher extracellular water percentage and CRP, and a higher prevalence of sarcopenic obesity (15.5%). These findings underscore that in older HD patients, malnutrition, inflammation, overhydration, and altered body composition frequently coexist, potentially amplifying mortality risk beyond that captured by BMI alone [33].

A recent systematic review and meta-analysis of 23 observational studies including 381,580 hemodialysis patients demonstrated that the association between obesity and mortality is highly dependent on the method of obesity assessment. While obesity defined by BMI was associated with lower all-cause mortality (RR 0.73, 95% CI 0.70–0.76), abdominal obesity assessed by waist circumference or waist-to-hip ratio increased mortality risk (RR 1.35, 95% CI 1.01–1.80), and studies using bioelectrical impedance analysis showed higher mortality in obese patients (RR 1.22, 95% CI 1.05–1.41). These findings reinforce that the so-called obesity paradox largely reflects limitations of BMI in capturing metabolically adverse fat distribution and body composition [34].

In this cohort of 679 patients undergoing maintenance hemodialysis, BMI was strongly associated with multiple demographic, biochemical, cardiovascular, and survival outcomes. The stratified analysis revealed substantial clinical heterogeneity across normal weight, overweight, and obese groups, underscoring the multidimensional implications of body size in chronic dialysis populations. Related to this issue, some of our findings from the results should be discussed here.

We found that normal-weight patients (BMI < 25 kg/m^2^) were younger and exhibited longer dialysis exposure, whereas increasing BMI was associated with progressively shorter dialysis vintage. In a prospective dialysis cohort, obesity was associated with a markedly higher mortality risk in patients younger than 65 years, whereas this association was largely attenuated in older patients, suggesting that the adverse impact of obesity may be substantially stronger in younger than in elderly dialysis populations [35]. In contrast, a large retrospective cohort of incident hemodialysis patients demonstrated an overall survival benefit with increasing BMI, which persisted in patients older than 65 years, while among younger patients, no significant differences in mortality across BMI categories were observed [36]. Taken together, large observational data indicate that the association between BMI and mortality in hemodialysis patients is modified by age and dialysis vintage, with the magnitude and direction of the BMI–mortality relationship varying across populations; in particular, the survival benefit of higher BMI appears more pronounced in incident patients, whereas additional increases in BMI confer little incremental benefit in older individuals [37]. Younger (but not young) HD patients at initiation may have a different comorbidity profile with less traditional, age-related comorbidities (as it was our case: significantly less CAD 64.4% vs. 78.1% in overweight and 76.8% in obese; less LVH 71.3% vs. 80.8% in obese; less PVD 24 vs. 33.6 in obese; less DM 15.7% vs. 28.8% in overweight and 37.6% in obese). Another explanation could be the fact that in a longer dialysis therapy duration, they lost weight and became normal-weight patients from overweight patients.

Dialysis adequacy also varied across BMI categories, with obese individuals demonstrating lower eKt/V values. As it is well known, Kt/V equations based on urea kinetics are not the ideal tools to evaluate dialysis adequacy in obese patients since the “V” (volume of distribution of urea) may be increased with increased body size. Therefore, Kt/V usually underestimates dialysis adequacy in these patients. That is why, trying to increase reliability, we are using in our centers equilibrated Kt/V (eKt/V) (correcting urea rebound) determined by OCM (online clearance monitoring by Adimea systems) and monitoring clinical and nutritional markers (absence of uremic symptoms, albuminemia, or achieving ultrafiltration goals). In our cohort, lower eKt/V (<1.2) remained independently associated with higher mortality, despite similar hemoglobin and serum albumin levels and the absence of uremic symptoms, underscoring the need for individualized dialysis prescriptions in obese patients.

Markers of iron metabolism differed significantly by BMI. The normal-weight patients had higher ferritin and transferrin saturation compared with both overweight and obese individuals, a pattern not attributable to differences in hemoglobin or inflammation. The higher CRP levels observed in overweight patients did not extend to the obese group, indicating a non-linear relationship between BMI groups and systemic inflammation. In a large cohort, obese individuals Yanoff demonstrated significantly lower serum iron (75.8 ± 35.2 vs. 86.5 ± 34.2 g/dL), higher ferritin (81.1 ± 88.8 vs. 57.6 ± 88.7 mg/L), elevated transferrin receptor (22.6 ± 7.1 vs. 21.0 ± 7.2 nmol/L), and markedly higher CRP (0.75 ± 0.67 vs. 0.34 ± 0.67 mg/dL), with obesity conferring a higher prevalence of iron deficiency by serum iron and transferrin receptor criteria. These discrepancies likely reflect differences in inflammation burden, iron supplementation practices, and dialysis-related iron handling across populations [38].

Cardiovascular comorbidities displayed clear BMI-dependent gradients. Overweight and obese patients had a higher prevalence of coronary artery disease, and peripheral vascular disease was also more frequent in obese individuals. Although left ventricular hypertrophy approached significance and left ventricular ejection fraction was preserved across BMI strata, the higher prevalence of atherosclerotic disease in higher BMI categories aligns with known metabolic and hemodynamic consequences of adiposity. Mineral metabolism markers also varied across categories, with obese patients exhibiting higher phosphate and calcium-phosphate product levels, findings that may have implications for long-term cardiovascular burden. In the obese group, higher phosphate levels could also be related to higher protein intake.

Metabolic and infectious comorbidities followed divergent patterns. Diabetes mellitus increased markedly with rising BMI, whereas hepatitis C was more common in the normal-weight individuals, suggesting differing etiological pathways and possibly reflecting distinct historical exposures or clinical phenotypes. Mortality did not differ significantly in the crude BMI comparisons; however, more nuanced findings emerged in survival analyses.

Though supported by large cohort analysis papers and some meta-analyses, the “obesity paradox” still remains a debated subject. A large retrospective analysis from the DaVita cohort, including 123,383 hemodialysis patients, reported a progressive reduction in all-cause, cardiovascular, and infection-related mortality with increasing time-averaged BMI, particularly among individuals younger than 65 years. In older patients (≥65 years), overweight and obesity were still associated with lower mortality compared to lower BMI, although further increases beyond 25 kg/m^2^ conferred no additional survival benefit. The survival advantage of higher BMI was most pronounced in incident hemodialysis patients, whereas individuals with longer dialysis vintage experienced a less marked effect [37]. One should also note the fact that the 25 kg/m^2^ threshold from the above study is considered the upper limit of normal BMI in the current classifications. In the DOPPS analysis of 9714 hemodialysis patients, higher BMI was consistently associated with lower death risk irrespective of age [7]. Ladhani [12] performed a meta-analysis including 852,162 patients on hemodialysis from 65 cohorts and reported that each 1 kg/m^2^ increase in BMI was associated with a 3% and 4% reduction in all-cause and cardiovascular mortality, respectively. Other cohorts have reported contrasting results. In NECOSAD-2, which followed 722 hemodialysis patients aged 50–75 years for 2 to 7 years, both low BMI (<18.5 kg/m^2^) and high BMI (≥30 kg/m^2^) were associated with increased mortality compared with normal BMI (22–25 kg/m^2^), with the excess risk greatest among underweight patients [39]. These data have been confirmed by a more recent NECOSAD analysis of 1,749 patients with 7 years of follow-up that found a U-shaped association in individuals younger than 65 years, where BMI < 18.5 or ≥30 kg/m^2^ increased mortality risk two-fold and 1.57-fold, respectively, while no association was observed among patients aged ≥65 years [35]. Delautre, in a large European cohort of 753 hemodialysis patients, found that metabolic syndrome driven primarily by abdominal obesity was strongly associated with major adverse cardiovascular events (OR 1.85, 95% CI 1.24–2.75), supporting the notion that adiposity-related metabolic disturbances remain clinically relevant despite the so-called obesity paradox [40]. As in the general population, using BMI alone in dialysis patients cannot accurately reflect abdominal fat deposition or differentiate between metabolically healthy and unhealthy body composition [41].

In our study the multivariable Cox regression model demonstrated that obesity, but not overweight, was independently associated with increased mortality after adjustment for age, sex, dialysis adequacy, biochemical parameters, cardiac function, and comorbidities. This association, though modest, contrasts with reports describing an “obesity paradox” in dialysis, whereby higher BMI has been associated with improved survival. The present findings suggest that the survival advantage of higher body mass may not extend uniformly across all BMI ranges, and that obesity, particularly in the context of elevated inflammation or cardiovascular disease burden, may confer additional risk. Kaplan–Meier survival curves further illustrated a stepwise reduction in median survival with increasing BMI. The normal-weight individuals demonstrated the longest survival, followed by the overweight and obese patients, with differences confirmed by log-rank testing. These results complement the multivariable findings and highlight the complex interplay between body composition and comorbid diseases. Our results are more in line with studies that have incorporated both BMI and WC, like a large Korean cohort study including 18,699 adult hemodialysis patients with 4 years of follow-up. They found that participants with the highest WC had a significantly higher risk of mortality, and that abdominal obesity could coexist across a wide range of BMI values [25].

Taken together, this study provides a comprehensive characterization of BMI-associated clinical phenotypes in hemodialysis and identifies obesity as a risk marker rather than a protective factor in this cohort. The findings reinforce the importance of individualized assessment of nutritional status, inflammation, dialysis dosing, and cardiometabolic risk when interpreting BMI in the dialysis population. Prospective studies integrating body composition metrics, inflammatory biomarkers, and longitudinal treatment data are warranted to further clarify the mechanisms underpinning these associations.

## 5. Limitations

This study has several strengths, including a well-characterized cohort of maintenance hemodialysis patients; comprehensive clinical and biochemical data; and robust multivariable analyses incorporating dialysis adequacy, cardiovascular comorbidities, and inflammatory markers. The inclusion of sensitivity analyses and interaction testing further strengthens the validity and robustness of the observed associations.

Several limitations should also be acknowledged: First, the relatively modest sample size, particularly the smaller proportion of obese patients, may have limited statistical power for certain subgroup and interaction analyses and may contribute to imbalance across BMI categories. Nevertheless, this cohort represents a multicenter, nationally diverse sample reflecting real-world hemodialysis practice in Romania. In addition, the observational design precludes causal inference and may be subject to selection and survivor bias, particularly in light of the observed differences in dialysis vintage across BMI categories. The normal-weight patients exhibited longer dialysis exposure, raising the possibility that long-term survivors may have experienced progressive weight loss due to protein–energy wasting, chronic inflammation, or intercurrent illness. Consequently, reverse causality cannot be excluded, whereby prolonged survival leads to lower body weight rather than lower body weight conferring a survival advantage.

Second, obesity was defined using body mass index, which does not distinguish between fat mass and lean mass and may misclassify patients with sarcopenic obesity or excess visceral adiposity. The absence of direct body composition measurements—such as imaging-based assessments or waist circumference—limits our ability to evaluate the specific contribution of muscle mass, visceral fat, and myosteatosis to mortality risk. Although inflammatory markers such as C-reactive protein, ferritin, and albuminemia were included in the analyses, residual confounding related to chronic inflammation cannot be fully excluded.

Third, obese patients exhibited lower delivered eKt/V, reflecting the well-recognized technical challenges of achieving adequate solute clearance in individuals with larger body size. Although adjustment for dialysis adequacy attenuated the association between obesity and mortality, it did not abolish it, suggesting that relative under-dialysis may contribute to, but does not fully explain, the excess risk associated with obesity. Moreover, dialysis session duration was unavailable, precluding calculation of ultrafiltration rate normalized to treatment time, which may better capture hemodynamic stress.

Finally, cause-specific mortality data were not available, limiting our ability to distinguish between cardiovascular and non-cardiovascular causes of death. As a result, we could not determine whether the excess mortality observed in obese patients with preserved left ventricular ejection fraction was driven primarily by cardiovascular events, infectious complications, or other mechanisms.

These limitations should be considered when interpreting our findings. Future prospective studies incorporating longitudinal body composition assessment, time-updated dialysis parameters, inflammatory profiling, and cause-specific outcomes are warranted to better elucidate the pathways linking obesity to adverse outcomes in the hemodialysis population.

## 6. Conclusions

In this multicenter cohort of Romanian hemodialysis patients, obesity defined by BMI was independently associated with higher all-cause mortality, while overweight showed no clear survival advantage compared with normal weight. Survival decreased stepwise from normal weight to overweight and was lowest in obese patients, contradicting the traditional obesity paradox described in many dialysis cohorts.

## Figures and Tables

**Table 1:** Baseline demographic, clinical, biochemical, and cardiovascular characteristics of the hemodialysis cohort stratified by BMI categories.

Variable	BMI < 25 kg/m^2^	BMI 25–30 kg/m^2^	BMI > 30 kg/m^2^	Total	*p*: <25 vs. 25–30	*p*: <25 vs. >30
Age (yr)	55.6 ± 14.4	59.5 ± 10.9	58.0 ± 10.0	57.2 ± 12.9	0.000	0.036
Male sex, n/N (%)	207/358 (57.8%)	128/196 (65.3%)	66/125 (52.8%)	401/679 (59.1%)	0.102	0.347
Dialysis vintage (yr)	5.1 ± 4.5	4.1 ± 4.0	3.2 ± 2.7	4.5 ± 4.1	0.005	0.000
Hb (g/dL)	11.3 ± 1.5	11.2 ± 1.3	11.4 ± 1.2	11.3 ± 1.4	0.420	0.741
Ferritin (ng/mL)	608.5 ± 484.6	511.7 ± 367.9	474.0 ± 297.0	555.9 ± 426.7	0.009	0.000
CRP (mg/L)	7.1 ± 12.8	11.1 ± 24.4	8.7 ± 13.5	8.5 ± 17.1	0.034	0.254
Albumin (g/dL)	4.1 ± 0.5	4.1 ± 0.9	4.1 ± 0.4	4.1 ± 0.6	0.386	0.440
Body weight (kg)	60.9 ± 8.9	79.6 ± 7.2	98.3 ± 14.6	73.2 ± 17.4	0.000	0.000
eKt/V	1.4 ± 0.3	1.4 ± 0.4	1.3 ± 0.4	1.4 ± 0.4	0.040	0.000
BMI (kg/m^2^)	21.5 ± 2.3	27.6 ± 1.4	34.71 ± 4.06	26.1 ± 12.7	0.001	0.001
Ultrafiltration (mL/session)	2096.9 ± 1412.1	2359.8 ± 1351.4	2439.6 ± 1364.7	2236.7 ± 1392.0	0.032	0.018
Calcium (mg/dL)	8.7 ± 0.9	8.7 ± 0.8	8.8 ± 0.9	8.7 ± 0.8	0.700	0.467
Phosphate (mg/dL)	4.6 ± 1.4	4.8 ± 1.4	5.1 ± 1.5	4.8 ± 1.5	0.292	0.002
Ca × PO_4_ (mg^2^/dL^2^)	40.5 ± 13.4	41.8 ± 14.0	45.2 ± 14.8	41.7 ± 14.0	0.303	0.002
Bicarbonate (mmol/L)	22.5 ± 4.0	22.5 ± 3.3	22.5 ± 6.9	22.5 ± 4.5	0.832	0.966
iPTH (pg/mL)	571.0 ± 604.9	629.2 ± 683.8	629.8 ± 781.1	598.6 ± 663.0	0.319	0.445
TSAT (%)	33.7 ± 15.4	30.0 ± 14.1	28.7 ± 11.2	31.7 ± 14.5	0.004	0.000
Coronary artery disease, n/N (%)	228/354 (64.4%)	153/196 (78.1%)	96/125 (76.8%)	477/675 (70.7%)	0.001	0.011
Heart valve calcifications, n/N (%)	202/326 (62.0%)	113/187 (60.4%)	73/120 (60.8%)	388/633 (61.3%)	0.778	0.827
Left ventricular hypertrophy, n/N (%)	233/327 (71.3%)	143/188 (76.1%)	97/120 (80.8%)	473/635 (74.5%)	0.258	0.052
LVEF (%)	57.7 ± 8.6	56.5 ± 9.7	56.6 ± 8.6	57.2 ± 9.0	0.160	0.242
Peripheral vascular disease, n/N (%)	86/358 (24.0%)	60/196 (30.6%)	42/125 (33.6%)	188/679 (27.7%)	0.107	0.045
Cerebrovascular disease, n/N (%)	73/358 (20.4%)	41/196 (20.9%)	21/125 (16.8%)	135/679 (19.9%)	0.913	0.432
Diabetes mellitus, n/N (%)	52/332 (15.7%)	51/177 (28.8%)	41/109 (37.6%)	144/618 (23.3%)	0.001	0.000
Hepatitis B, n/N (%)	24/357 (6.7%)	11/196 (5.6%)	11/125 (8.8%)	46/678 (6.8%)	0.716	0.429
Hepatitis C, n/N (%)	93/358 (26.0%)	35/196 (17.9%)	14/125 (11.2%)	142/679 (20.9%)	0.035	0.000
Mortality, n/N (%)	185/358 (51.7%)	108/196 (55.1%)	67/125 (53.6%)	360/679 (53.0%)	0.477	0.755

yr = years; Hb = hemoglobin; CRP = C-reactive protein; eKt/V = equilibrated Kt/V; Ca × PO_4_ = calcium–phosphate product; iPTH = intact parathyroid hormone; TSAT = transferrin saturation; LVEF = left ventricular ejection fraction. Values are mean ± SD for continuous variables and n/N (%) for categorical variables.

**Table 2:** Univariate and multivariate Cox proportional hazards models for all-cause mortality in haemodialysis patients.

Variables	Univariate HR (95% CI)	Univ *p*	Multivariate HR (95% CI)	Multiv *p*
Age (years)	1.037 (1.028–1.046)	<0.001	1.042 (1.031–1.052)	<0.001
Male vs. female	1.204 (0.972–1.492)	0.090	0.945 (0.74–1.208)	0.653
Overweight vs. normal weight	1.185 (0.944–1.488)	0.143	1.022 (0.781–1.339)	0.873
Obese vs. normal weight	1.557 (1.188–2.04)	0.001	1.411 (1.008–1.976)	0.045
LVEF 41–49% vs. LVEF < 35%	1.278 (0.982–1.664)	0.068	0.725 (0.489–1.075)	0.110
LVEF ≥ 50% vs. LVEF < 35%	0.677 (0.544–0.842)	<0.001	0.665 (0.482–0.918)	0.013
Albumin < 4.0 g/dL	1.536 (1.247–1.892)	<0.001	1.198 (0.931–1.541)	0.160
Hemoglobin < 10 g/dL	1.164 (0.889–1.524)	0.270	1.155 (0.854–1.563)	0.350
Ferritin < 200 or >500 ng/mL	0.833 (0.672–1.033)	0.096	0.749 (0.589–0.952)	0.018
CRP > 0.5 mg/dL	1.9 (1.418–2.546)	<0.001	1.781 (1.289–2.461)	<0.001
Phosphate > 5.5 mg/dL	0.785 (0.619–0.995)	0.045	0.85 (0.651–1.108)	0.229
iPTH < 150 or >600 pg/mL	0.843 (0.684–1.039)	0.110	0.879 (0.699–1.107)	0.274
Coronary artery disease (yes vs. no)	1.261 (0.989–1.608)	0.062	0.789 (0.582–1.068)	0.125
Peripheral vascular disease (yes vs. no)	1.351 (1.084–1.685)	0.007	0.903 (0.677–1.205)	0.488
Cerebrovascular disease (yes vs. no)	1.475 (1.163–1.872)	0.001	1.032 (0.766–1.39)	0.837
Diabetes mellitus (yes vs. no)	1.879 (1.456–2.424)	<0.001	1.775 (1.338–2.355)	<0.001
Ultrafiltration > 500 mL/session	0.626 (0.449–0.874)	0.006	0.702 (0.481–1.026)	0.067
eKt/V < 1.2	1.511 (1.203–1.899)	<0.001	1.343 (1.031–1.749)	0.029

## Data Availability

The data presented in this study are available upon reasonable request from the corresponding author. The data are not publicly available due to privacy and ethical restrictions.
